# Theoretical Evaluation of the Impact of Hyperthermia in Combination with Radiation Therapy in an Artificial Immune—Tumor-Ecosystem

**DOI:** 10.3390/cancers13225764

**Published:** 2021-11-17

**Authors:** Stephan Scheidegger, Sergio Mingo Barba, Udo S. Gaipl

**Affiliations:** 1 ZHAW School of Engineering, Zurich University of Applied Sciences, 8401 Winterthur, Switzerland; ming@zhaw.ch; 2 Faculty of Science and Medicine, University of Fribourg, 1700 Fribourg, Switzerland; 3 Translational Radiobiology, Department of Radiation Oncology, Universitätsklinikum Erlangen, 91054 Erlangen, Germany; Udo.Gaipl@uk-erlangen.de

**Keywords:** systems medicine, immune system in silico, perceptron, antigen pattern, danger signal, fractionation, immune response

## Abstract

**Simple Summary:**

Radio-sensitizing effects of moderate or mild hyperthermia (heating up tumor cells up to 41–43 °C) in combination with radiotherapy (thermoradiotherapy) have been evaluated for decades. However, how this combination might modulate an anti-tumor immune response is not well known. To investigate the dynamic behavior of immune–tumor ecosystems in different scenarios, a model representing an artificial adaptive immune system in silico is used. Such a model may be far removed from the real situation in the patient, but it could serve as a laboratory to investigate fundamental principles of dynamics in such systems under well-controlled conditions and it could be used to generate and refine hypothesis supporting the design of clinical trials. Regarding the results of the presented computer simulations, the main effect is governed by the cellular radio-sensitization. In addition, the application of hyperthermia during the first radiotherapy fractions seems to be more effective.

**Abstract:**

There is some evidence that radiotherapy (RT) can trigger anti-tumor immune responses. In addition, hyperthermia (HT) is known to be a tumor cell radio-sensitizer. How HT could enhance the anti-tumor immune response produced by RT is still an open question. The aim of this study is the evaluation of potential dynamic effects regarding the adaptive immune response induced by different combinations of RT fractions with HT. The adaptive immune system is considered as a trainable unit (perceptron) which compares danger signals released by necrotic or apoptotic cell death with the presence of tumor- and host tissue cell population-specific molecular patterns (antigens). To mimic the changes produced by HT such as cell radio-sensitization or increase of the blood perfusion after hyperthermia, simplistic biophysical models were included. To study the effectiveness of the different RT+HT treatments, the Tumor Control Probability (TCP) was calculated. In the considered scenarios, the major effect of HT is related to the enhancement of the cell radio-sensitivity while perfusion or heat-based effects on the immune system seem to contribute less. Moreover, no tumor vaccination effect has been observed. In the presented scenarios, HT boosts the RT cell killing but it does not fundamentally change the anti-tumor immune response.

## 1. Introduction

Preclinical and, to some extent, clinical research demonstrated that radiotherapy (RT) is able to modulate anti-tumor immune responses [1,2,3,4]. The idea of activating the immune system by radiation leads to the question of how hyperthermia (HT) in combination with RT could help to trigger or amplify such an anti-tumor response.

Radio-sensitizing effects of HT in combination with RT (thermoradiotherapy, HT-RT) have been evaluated for decades. Effects have been investigated on molecular [5,6,7], cellular [8,9], and tissue scale [10,11,12]. Regarding the tissue level, increased perfusion leading to a removal of acidic metabolites [13,14,15] and re-oxygenation [16,17,18] have been discussed by several authors. Re-oxygenation is known as a radio-sensitizing factor [19,20], but the effect of, e.g., combining 3–6 of total 32 fractions of RT with HT may be very limited [21], especially when considering time gaps between application of HT and RT of 30–120 or more minutes in clinical routine treatments. However, the wash-out of acidic metabolites by increased perfusion below 42–43 °C could improve the immune system response [22,23,24,25]. In addition, increased perfusion may improve the accessibility for immune cells, leading to a better detection of antigenic patterns and enhanced tumor–immune cell interaction via related to danger signals such as Heat Shock Proteins (HSP) [26,27,28]. There seem to be many contributing factors and it is difficult to identify the key processes leading to the clinically observed improved therapy outcome of HT-RT. Whereas on the cellular level, more or less controlled experiments in vitro may help to understand molecular or cellular aspects of the additive or synergistic heat- and radiation-induced responses, the dynamic interaction of the immune system with the tumor tissue is patient-specific and would require a time-resolved monitoring of immune cell activity in the body or at least, in the tumor environment. This information is hard to access during clinical trials since, for example, repeated (frequent) biopsy material has to be sampled from the patients and analyzed.

However, treatment optimization would require a profound understanding of the dynamic response of tumor and host tissue as well as the immune system. Whereas clinical trials may generate knowledge about the effectiveness of specific aspects such as fractionation schemes and can be seen as acid tests for novel approaches for anti-cancer treatments, the investigation of the dynamic behavior should include the analysis of time-resolved data representing the complex interaction in the tumor-host-immune system. Such a tumor-host-immune system may be considered as an ecosystem [29,30,31]. This may include the interaction between sub-populations of tumor cells, tumor-associated cells (e.g., fibroblasts), host tissue, endothelial cells, and immune cells. Understanding the dynamics in such a complex ecosystem may be pivotal as soon as the therapy outcome is governed by the dynamic interactions between the different actors in the system. Regarding the immune system as a part of the whole, the complexity is enormous since not only the immune cells (e.g., T-cells or macrophages) in the tumor compartment but the systemic response has to be considered as well [32].

At a first glance, there seems to be no way to get a profound insight into the complex dynamic interactions in such an immune–tumor ecosystem. Regarding the effects of HT, the processes taking place on different scale levels may influence the system in an obscure manner, but the identification of key processes would support the optimization of hyperthermia in combination with RT (e.g., timing of HT sessions and RT fractions). The different therapy regimens may be tested in clinical trials. Mathematical models and computer simulations could be used to guide the search for optimal conditions for HT-RT. The complexity will probably hamper the development of predictive models covering all the aspects relevant for therapy response in vivo or in patient. The situation may be different as soon as not prediction is sought. Artificial immune–tumor ecosystems may be far removed from the real situation in the living organism, but they could serve as a laboratory to investigate fundamental principles of dynamics in such systems under well-controlled conditions. As a complementary approach to biological experiments in vitro, ex vivo, in vivo, or clinical trials, such sandbox games could be used to generate and refine hypothesis supporting the design of clinical trials. Scheidegger et al. [33] proposed an artificial immune–tumor model system covering two essential aspects: ecosystem dynamics between host tissue and different tumor sub-clones and antigen pattern recognition by a learning (adaptive) immune system. The proposed model exhibited some interesting aspects: as a response to radiation treatments, host tissue becomes immune-suppressive whereas the tumor-related response is improved by the re-growing tumor cell populations and subsequent necrosis. This behavior is dependent on the interaction strength (competition) between the host tissue and the different tumor sub-populations. Regarding these results, an interesting question is whether there are parameters influencing the specific anti-tumor immune response in this model which are related to effects of HT. Therefore, the purpose of this study is to identify such model parameters and to investigate the potential effect of combining HT sessions with different RT fractionation schemes in the framework of the proposed artificial system. In contrast to other mathematical models for immune–tumor systems [1,34], we consider the adaptive immune system as a trainable (programmable) unit and anti-cancer treatments as means to train the immune system to battle against cancer.

## 2. Materials and Methods

**Figure 1:**
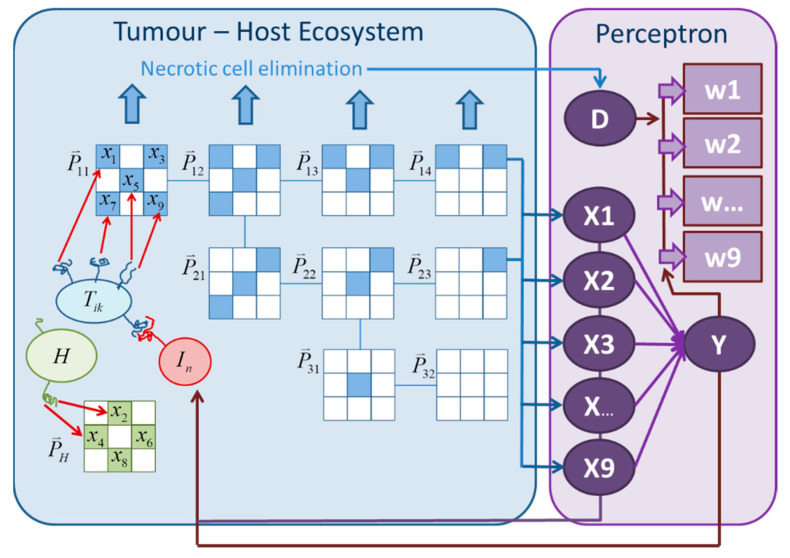
Structure of the tumor–immune system model and mutation tree/tumor sub-clones with associated antigen pattern vectors ${\vec{P}}_{ik}$. Vector components may represent epitopes on a specific complex protein or may be distributed over different proteins. According to the presence antigen vector components, an antigen signal $X_{n}$ together with the danger signal *D* generate a perceptron response Y which induces the growth and immigration of effector cells (*I_n_*).

The artificial immune–tumor ecosystem proposed by Scheidegger et al. [33] consists of two major components: a tumor ecosystem, including host tissue and immune cells in the tumor compartment, and a perceptron [35] for antigen pattern recognition (Figure 1). The idea of using a perceptron to mimic the immune system’s ability of pattern recognition is based on the danger model proposed by P. Matzinger [36]. Following this concept, the immune system is only activated when a danger signal and antigens are coincidently present (adjuvanticity plus antigenicity). The proposed model uses a very simplistic approach for danger signal generation, which is assumed to be proportional to the amount of necrotic or immune system-activating apoptotic cells [37,38,39]. In the following, the model equations are presented (a detailed explanation of the model is given by Scheidegger et al. [33]). The dynamic interaction between the different tumor sub-clones $T_{ik}$ and the host tissue *H* is given by the following system of ordinary differential equations:

(1) $$\begin{array}{l} \frac{dT_{11}}{dt}=\left(k_{T11}-k_{mut}-k_{eT}-r_{11}k_{IT}-k_{HT}H-k_{TT}T-(\alpha_{T}+2\beta_{T}\Gamma )\cdot R\right)\cdot T_{11}\\ \frac{dT_{ik}}{dt}=\left(k_{Tik}-k_{eT}-r_{ik}k_{IT}-k_{HT}H-k_{TT}T-(\alpha_{T}+2\beta_{T}\Gamma )\cdot R\right)\cdot T_{ik}+k_{mut}\cdot q_{il}T_{lk}\\ \frac{dH}{dt}=\left(k_{aH}-k_{eH}-r_{H}k_{IH}-k_{bH}H-k_{TH}T-(\alpha_{H}+2\beta_{H}\Gamma )\cdot R\right)\cdot H \end{array}$$

where $k_{Tik}\cdot T_{ik}$ is the reproduction rate of the tumor sub-population $T_{ik}$ (the tumor sub-clones are assumed to form a mutation tree with branches *k*; $k_{T11}\cdot T_{11}$ denotes the reproduction rate of the population *i* = 1 and *k* = 1, for the host tissue, the corresponding rate is $k_{aH}\cdot H$); $k_{eT}\cdot T_{ik}$ (and $k_{eH}\cdot H$ for host tissue) represents the rate of cell elimination (death rate) independent from radiation-induced cell killing and immune system-mediated cell elimination; the immune system-related elimination rate is calculated by $r_{ik}k_{IT}\cdot T_{ik}$ with an interaction coefficient $k_{IT}$ (assumed to be the same for all tumor sub-clones, $r_{ik}$ defines the match with antigen-receptor binding sites and will be explained later; for host tissue, a different coefficient $k_{IH}$ is used); $k_{mut}\cdot q_{il}T_{lk}$ gives the rate of mutation ($q_{il}$ is a matrix representing the topology of the population network, see [33]). Competition between the different tumor sub-populations is included by $k_{TT}T\cdot T_{ik}$ (with the total amount of tumor cells *T*) and for host tissue by $k_{TH}T\cdot H$; $k_{bH}\cdot H^{2}$ represents the self-inhibition of host tissue growth. For radiation-induced cell killing, a dynamic linear-quadratic law with a transient biological dose equivalent Γ [40] is used. The radiation-induced death rate is dependent on the radiation dose rate *R*, the radio-sensitivity coefficients $\alpha_{H}$ and $\beta_{H}$ for host and $\alpha_{T}$ and $\beta_{T}$ for tumor cells (in this study assumed to be the same for all tumor sub-clones). The transient biological dose equivalent Γ is rising with the dose rate *R* and decaying with a repair constant γ:

(2) $$\frac{d\Gamma_{T,H,I}}{dt}=R-\gamma_{T,H,I}\Gamma_{T,H,I}$$

The indices are indicating that—depending on the cellular repair capability–different repair rate constants γ have to be applied for tumor cells, host tissue, and immune cells (effector cells, the exchange of these cells in the tumor compartment leads to a certain “repair” effect which depends on the immigration speed of these cells).

The different cell death processes will lead to apoptotic and necrotic cells. Apoptotic cell death seems not to be equally considered as a danger signal compared to necrotic cell death [41], where the release of intracellular Heat Shock Proteins (HSP’s) may be involved [28]. Apoptosis may generate danger signals [42,43,44] but this usually happens in particular situations and they can be pro- or anti-inflammatory [45,46]. In the presented model, we distinguish only between immune-stimulating and non-stimulating cell elimination processes. As immune-stimulating cell death processes, inflammatory processes, necrotic cells, non-cleared apoptotic cells which undergo secondary necrosis, or immunogenic apoptosis can be seen as immune-stimulating processes [47] and will contribute to the danger signal. The calculation of this signal is based on the amount of these cells which are “transformed” damaged pre-immune-stimulatory tumor cells *N*_*p*,*ik*_ and damaged pre-immune-stimulatory host tissue cells *N*_*p*,*H*_. Only host tissue cells are considered to be able to undergo a non-immune-stimulatory elimination pathway (e.g., apoptotic cell death processes that are characterized by dying cells with still intact membrane integrity and that do not generate any danger signal) by the rate $k_{ap}N_{p,H}$:

(3) $$\begin{array}{l} \frac{dN_{p,11}}{dt}=\left(k_{eT}+r_{11}k_{IT}+(\alpha_{T}+2\beta_{T}\Gamma )\cdot R\right)\cdot T_{11}-k_{pn}N_{p,11}\\ \frac{dN_{p,ik}}{dt}=\left(k_{eT}+r_{ik}k_{IT}+(\alpha_{T}+2\beta_{T}\Gamma )\cdot R\right)\cdot T_{ik}-k_{pn}N_{p,ik}\\ \frac{dN_{p,H}}{dt}=\left(k_{eH}+r_{H}k_{IH}+(\alpha_{H}+2\beta_{H}\Gamma )\cdot R\right)\cdot H-(k_{pn}+k_{ap})\cdot N_{p,H} \end{array}$$

According Equations (1) and (3), only the elimination processes related to radiation, immune system-mediated response, and other cell death described by the death rate parameters $k_{eT}$ and $k_{eH}$ are considered to produce dying cells, which subsequently are transformed to immune-stimulatory necrotic or apoptotic cells at the rate $k_{pn}N_{n,ik}$ and $k_{pn}N_{n,H}$. These cells are calculated by:

(4) $$\begin{array}{l} \frac{dN_{11}}{dt}=k_{pn}N_{p,11}-k_{n}N_{11}\\ \frac{dN_{ik}}{dt}=k_{pn}N_{p,ik}-k_{n}N_{ik}\\ \frac{dN_{H}}{dt}=k_{pn}N_{p,H}-k_{n}N_{H} \end{array}$$

In summary—and in contrast to the model presented by Scheidegger et al. [33]—the danger signal generation includes a two-step process with lethally damaged cells which subsequently transforms to “immune-system-activating” cells as described above. For calculating the danger signal, a sigmoidal relationship between the signal strength and the amount of dying cells is assumed:

(5) $$D=\frac{{\left[\sum_{i,k}N_{ik}+N_{H}\right]}^{2}}{L_{act}^{2}+{\left[\sum_{i,k}N_{ik}+N_{H}\right]}^{2}}$$

$L_{act}$ governs the steepness of this sigmoidal relation between the amount of immune-stimulatory necrotic or apoptotic cells and the *D* (activation response).

The task of the adaptive immune system in principle is the detection of antigen patterns and a response generation based on the presence of the danger signal *D*. To mimic this process, Scheidegger et al. [33] proposed a perceptron as a structure which enables the immune system’s adaptability and ability to learn, along with molecular danger signals and antigen-antibody (or antigen-receptor) interactions. For this, an antigen pattern vector $\vec{X}=X_{i}$ can be defined. Every cell of a specific population (tumor sub-clones and host tissue) bears a corresponding pattern, which is defined by the elements of the antigen pattern vector. The presence of a component of the pattern vector (molecular signal) is considered to be dependent on the number of cells bearing this specific component. According to the pattern used in this study (Figure 1), the antigen signal strength of the first component for example is given by:

(6) $$X_{1}=\frac{{\left({\tilde{T}}_{11}+{\tilde{T}}_{12}+{\tilde{T}}_{13}+{\tilde{T}}_{14}\right)}^{2}}{{\left(X_{act}\right)}^{2}+{\left({\tilde{T}}_{11}+{\tilde{T}}_{12}+{\tilde{T}}_{13}+{\tilde{T}}_{14}\right)}^{2}}$$

with ${\tilde{T}}_{ik}=T_{ik}+\eta N_{p,ik}+\chi N_{ik}$: pre-immune-stimulatory and immune-stimulatory necrotic or apoptotic cells are considered to contribute to the presence of antigens, but with the weighting factors η and χ. Similar to the sigmoidal relation in Equation (5), $X_{act}$ influences the activation response. Depending on the presence of a specific antigen signal, the perceptron is used to adapt corresponding antigen weights $w_{i}$ for generating the perceptron response by comparing the actual danger signal strength *D* with the perceptron response Y:

(7) $$\frac{dw_{i}}{dt}=a\cdot (D-Y)\cdot X_{i}$$

with the perceptron response $Y=\Sigma^{\xi }/(Y_{act}^{\xi }+\Sigma^{\xi })$ and $\Sigma =\sum_{i=1}^{9}w_{i}X_{i}$. Even here, the perceptron response is modelled by a sigmoidal function, whose shape can be adapted by the powers ξ and the activation response parameter $Y_{act}$.

The perceptron response Y directly governs the production effector cells by the production rate $k_{I}YX_{n}$. The presented model does not distinguish between the different immune-response pathways and is based on a simplistic elimination process, where the receptor binding of an effector cell of the population $I_{n}$ with a tumor cell bearing the corresponding antigen will contribute to the tumor cell elimination. The match of antigen pattern with the effector cell population vector $I_{n}=\vec{I}$ is evaluated by the dot product between $\vec{I}$ and an antigen pattern vector $\vec{P}$ with components = 1 for bearing a specific antigen corresponding to the antigen pattern vector component $X_{n}$ and 0 otherwise: $r_{ik}=\vec{I}•{\vec{P}}_{ik}$. Finally, the elimination of effector cells is considered by the elimination rate constant $k_{eI}$ and the radiation-induced elimination by a TBDE-based LQ model with the radio-sensitivity coefficients $\alpha_{I}$ and $\beta_{I}$. At this point, it is important to keep in mind that only the immune cells in the tumor compartment are irradiated and that compared to the stem cells in the red bone marrow, the radio-sensitivity of these effector cells may be lower. The very simplistic concept used here may be more suitable for describing the T-cell–mediated response. Summing up these rates, the temporal change of the antigen or immune cell population can be calculated by:

(8) $$\frac{dI_{n}}{dt}=k_{I}YX_{n}-(k_{eI}+(\alpha_{I}+2\beta_{I}\Gamma_{I})\cdot R)\cdot I_{n}-k_{IT}\cdot {\left(\sum_{i,k}r_{ik}T_{ik}\right)}_{n}$$

The selection of parameter values (Table 1, Table 2 and Table 3) used in this study is representing a scenario where the radiation sensitivity of irradiated immune cells or antibodies in the tumor compartment are assumed to be less than the sensitivity of tumor cells but more than the host tissue. The repair parameter $\gamma_{I}$ in the kinetic model for $\Gamma_{I}$ (TBDE for effector cells, Equation (2)) is not only determined by the intrinsic repair of cells (if there is repair) but by the replacement of effector cells in the irradiated compartment. Therefore, the value for $\gamma_{I}$ should be above the one of $k_{eI}$. For the radio-sensitivity of tumor cells, a value close to colon cancer lines is used [48,49]. It is important to note here that the alpha and beta values cannot be directly compared with the standard LQ model since the kinetic model for the TBDE will reduce cell killing by repair. The effective alpha and beta values are therefore lower in this model (with $\gamma_{T}=3d^{-1}$: $\alpha_{T,eff}=0.128\;{\mathrm{Gy}}^{-1}$ and $\beta_{T,eff}=0.020\;{\mathrm{Gy}}^{-1}$), representing more radio-resistant tumor cells such as, e.g., cervix carcinoma cells.

The tumor and host tissue growth parameters have been selected based on the following criteria: the tumor is considered as a fast-growing tumor (doubling time of 20 days for all tumor sub-populations; $k_{Tik}=3.46\cdot 10^{-2}d^{-1}$), whereas the host tissue is assumed to repopulate slightly slower. The equilibrium level $H_{eq}$ for host tissue (homeostasis) is determined by the values of $k_{aH}$ and $k_{eH}$ to 250 (2.50 × 10^11^ cells). Assuming an average volume of 2 · 10^3^ μm^3^ per cell, the initial compartmental volume is 500 cm^3^. The equilibrium levels for host ($H_{eq}$) and tumor ($T_{eq}$) cell population can be calculated by the equilibrium conditions from Equation (1):

(9) $$T_{eq}=\frac{k_{Tik}-k_{eT}}{k_{TT}}\;\mathrm{and}\;H_{eq}=\frac{k_{aH}-k_{eH}}{k_{bH}}\;\mathrm{and}\;H_{eq}=\frac{k_{aH}-k_{eH}}{k_{bH}}$$

The equilibrium level for the tumor cell population without immunogenic elimination is set to 306 (3.06 × 10^11^ cells). This corresponds to a scenario where the tumor has less growth limitation than the host tissue.

As stated in the introduction, many processes may contribute to the effect of HT. Biophysical models may be used for the description of temperature-dependent effects such as inhibition of repair proteins or perfusion changes. Even non-thermal effects could be considered. It is important to clarify here that this study does not focus on the detailed mode of action of HT. The proposed model describes the tumor–host tissue evolution over about 5 years and focusses on large time scales. Therefore, a multi-scale approach including HT-effects in an implicit manner is used. The parameters in the following Section 2.1 and Section 2.2 are considered to be susceptible for hyperthermia.

### 2.1. Cellular Radiobiological Parameters

Assuming that tumor cells are radio-sensitized by heat-induced impair of the repair system [50,51,52], the radio-sensitivity parameters $\alpha_{T}$ and $\beta_{T}$ are modified according to the biophysical model proposed by van Leeuwen et al. [53]. The temperature during HT treatment (duration 60 min per session) was fixed to 42 °C and the time gap was assumed to be the same for all HT-RT treatments (30 min). Calculating the enhancement factor for the radio-sensitivity parameters for SiHa and HeLa cells using this model gives for both cell lines a similar value: $\alpha_{T}(42{}^{\circ}C)\approx 1.96\cdot \alpha_{T}(37{}^{\circ}C)$ and $\beta_{T}(42{}^{\circ}C)\approx 0.34\cdot \beta_{T}(37{}^{\circ}C)$. These are the values used in this study to mimic the effect of HT in combination with RT. In the dynamic LQ-model, an additional parameter for repair kinetics, $\gamma_{T}$ may be influenced by HT. In contrast to the well-established LQ formula, repair kinetics is separated from $\alpha_{T}$ and $\beta_{T}$; these coefficients can be considered to describe a baseline radio-sensitivity. Since the important aspect in the immune–tumor ecosystem model is the amount of radiation-induced necrotic cells, there is no principal difference in the effect when modifying only radio-sensitivity by $\alpha_{T}$ and $\beta_{T}$ instead of $\gamma_{T}$. Tumor tissue is assumed to have slow repair; therefore, this value was set to 3 d^−1^ for RT only (incomplete repair between the RT fraction; 10 d^−1^ corresponds to more or less complete repair between the RT fractions). Hyperthermia was assumed to reduce repair speed (repair protein inhibition) specifically for tumor cells. Therefore, we tested the sensitivity of the model to changes of $\gamma_{T}$. The effect of these variations is small and does not change the dynamics in the system. To keep the model simple, the full effect of HT was only considered by the given factors for $\alpha_{T}$ and $\beta_{T}$.

### 2.2. Parameters Influencing the Tumour–Immune System Interaction

Besides the radio-sensitivity parameters describing the cellular response to HT-RT (indirect immune activation via production of necrotic or immune-stimulatory apoptotic cells), thermal-induced modifications of immune system response are related to processes on cellular as well as tissue or systemic level. Thermally induced changes in perfusion and vascular permeability may enhance the accessibility of immune cells (not only effector cells) to the tumor compartment. To model the perfusion enhancement, the data from Song et al. [11,54] are used for a simple model: the perfusion enhancement factor *PEF* is calculated by a first order kinetic model: $d\theta /dt=k_{perf1}-k_{perf2}\cdot \theta$ with the condition $k_{perf1}/k_{perf2}$ = 1 and PEF=1+θ. This leads to a perfusion enhancement of a factor 2 which is reported by Song [11] for tumor tissue heated up to 42 °C. To achieve the temporal course of perfusion changes observed by Song et al. [54], the values for $k_{perf1}$ and $k_{perf2}$ are set to 200 d^−1^. According to the data from Song [54], modification of perfusion can be considered as a fast process, where during heating, perfusion increases to a factor 2 within 30–40 min and decreases within 30 min after heating to the baseline level.

In contrast to this fast process, a second slower process was included in the model to describe some “long-term” effects of HT. This model has the same structure but the rate parameters are set to lower values: $d\phi /dt=k_{ims1}-k_{ims2}\cdot \phi$. The values for these immune stimulating parameters ($k_{ims1}=7d^{-1}$ and $k_{ims2}=7d^{-1}$) are selected to mimic the experimental data for MHC class I antigen presentation after hyperthermia from Ito et al. [27], where rat T-9 glioma cells were heated up to 43 °C for one hour. According the data from Ito et al. [27], the enhancement of antigen expression starts 24 h after heating, reaches a maximum (two-fold increase) at 48 h after heating, and then decays to the baseline expression level cells at 72 h. To simulate this scenario, one day after a hyperthermia treatment the parameter $k_{ims1}$ was “switched on” for 24 h. The immune stimulation factor is defined by: ISF=1+ϕ.

Regarding the effect of perfusion, effector cells are considered to have a better accessibility to the tumor compartment. Since $k_{I}$ does not only describe the production rate of effector cells but includes migration speed to the irradiated compartment as well, this parameter is modified for HT by the perfusion enhancement factor: $k_{I,HT}=PEF\cdot k_{I}$.

The antigen pattern detectability (parameter *X_act_*) may be influenced by HT via in increased antigen presentation which is related to an enhanced recognition by the immune cells (macrophages, APCs). By decreasing the value for $X_{act}$, the signal “antigen present” will increase stronger (steeper slope) at small numbers of tumor cells bearing the corresponding antigen. In this model, the shift of $X_{act}$ is considered to be related with the slow process: $X_{act,HT}=X_{act}/ISF$.

The danger signal parameter $L_{act}$ can be used to describe HT-induced modifications of the danger signal generation. Regarding Equation (5), the danger signal in the proposed model is assumed to be dependent on the amount of immune-stimulatory necrotic or apoptotic cells. The amplification of this danger signal for example by excess HSP release is considered by varying parameter values of $L_{act}$. In analogy to $X_{act}$, this HT-related modification is assumed to be related to the slow process (more HSP production, lowering of $L_{act}$ shifts the response curve to the left: $L_{act,HT}=L_{act}/ISF$). For comparison, scenarios for both parameters have been studied for the fast (directly perfusion-related) process ($X_{act,HT}=X_{act}/PEF$; $L_{act,HT}=L_{act}/PEF$) as well.

### 2.3. Investigated Scenarios and Fractionation Schemes

In this study, nine antigen pattern components and nine tumor sub-clones according to Scheidegger et al. [33] were used. The structure of mutation tree is displayed in Figure 1.

**Figure 2:**
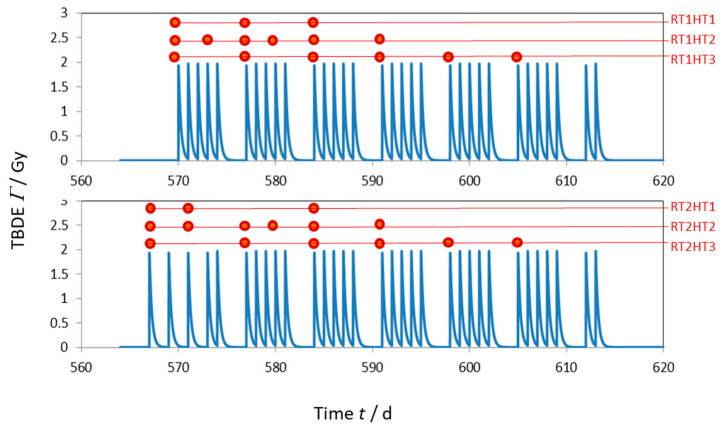
Different scenarios for fractionation and HT sessions: RT is applied in 32 fractions with 2 Gy per fraction, The HT scheme in RTxHT2 corresponds to the HYCAN trial [55], RTxHT3 to the KSA bladder trial [56].

Different fractionation schemes and combinations with HT have been evaluated (Figure 2). The tumor control probability TCP was calculated by the total amount of tumor cells *T*: $TCP=e^{-T}$. The TCP was evaluated at the time point with the lowest value of T during or after RT or HT-RT application. In the computer simulations, the artificial immune–tumor ecosystem evolved 560–570 days before irradiation. The total simulation time was set to 1800 days. For numerical integration, a Runge-Kutta algorithm with a time increment of *dt* = 10^−3^ d was selected.

## 3. Results

For the different treatment schemes displayed in Figure 2, the TCP has been calculated. The parameter values for the selected scenarios are adapted to achieve a baseline TCP of 0.8 without HT. In Table 4, the resulting TCP for the evaluated parameters are summarized. The highest TCP was achieved by RT1HT2 protocol (0.990) and a scenario where all HT-susceptible parameters where modified.

The range of TCP values for treatments with HT was 0.931–0.990. In general, the differences between the corresponding HT protocols for the two RT fractionation schemes (RT1 and RT2) are less than Δ*TCP* = 0.01 and clearly smaller than the impact of HT (TCP-rise of 0.130–0.192). Regarding the slow and fast process according to the HT models for $X_{act}$ and $L_{act}$ in Section 2.2, the perfusion-like process almost does not affect the TCP value while the slower process slightly improves it when it is applied to the $L_{act}$ parameter. However, the main improvement of the treatment outcome produced by hyperthermia is the cell radio-sensitization effect, i.e., the change in the $\alpha_{T}$ and $\beta_{T}$ parameters. For this reason, the HT1-Protocols have the lowest impact due to the smaller number of HT sessions.

**Figure 3:**
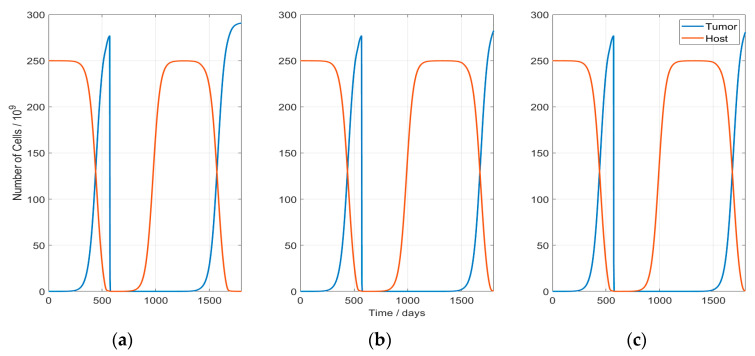
Development of the host—and tumor—cell populations: The sudden drop in the tumor population indicates the time point of RT start (day 570). After RT, the tumor starts to regrowth and approaches in every scenario the equilibrium level of 306 × 10^6^ cells. The scenarios presented here correspond to: (**a**) Case with no HT (RT1HT0); (**b**) Case RT1HT3; (**c**) Case RT1HT2.

In Figure 3, the resulting course of the host and tumor populations are shown. All the studied scenarios followed the same behavior with two tumor growth phases: the first one before RT and the second one after RT (tumor recurrence). Hyperthermia does not change qualitatively this course; however, it delays the second tumor regrowth by enhancing the radiation-induced cytotoxicity and the immune system response.

**Figure 4:**
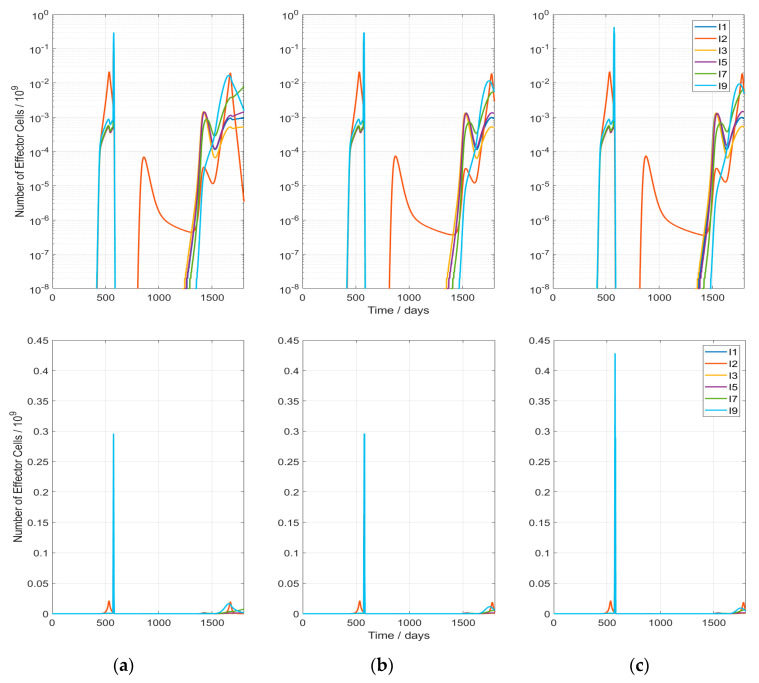
Development of effector immune cells in case of HT-induced modification of all parameters (last column in Table 4): For the host-related effector cells, only the population I2 (red line) is displayed; the other host-associated populations behave identically. The scenarios presented here correspond to (**a**) Case with no HT (RT1HT0); (**b**) Case RT1HT3; (**c**) Case RT1HT2; upper figures with logarithmic axis.

In Figure 4, the evolution of the effector cell populations is presented. Hyperthermia clearly increases the immune cells production during the first phase of treatment. However, no antitumor-vaccination effect is observed in any of the cases: This is clearly visible in the lower diagrams of Figure 4, where the immune cell numbers are plotted with a linear scale. In the upper part of Figure 4 (logarithmic plots), the weak responses during host and tumor regrowth become visible. A fundamental behavior of the system is visible during host tissue repopulation after treatment: In a first phase (around day 800), the host-related immune cell population (I2) rises, based on the previously evolved perceptron weights and the increasing presence of host tissue cells. Due to the lack of a danger signal during host tissue regrowth, the effector cell production and immigration drop after an initial rise. This is related to an evolution of perceptron weights (Equation (7)) to negative values. Comparing the three displayed scenarios, no substantial changes are observed between the different hyperthermia schemes, so the immune response is similar in all the cases.

**Figure 5:**
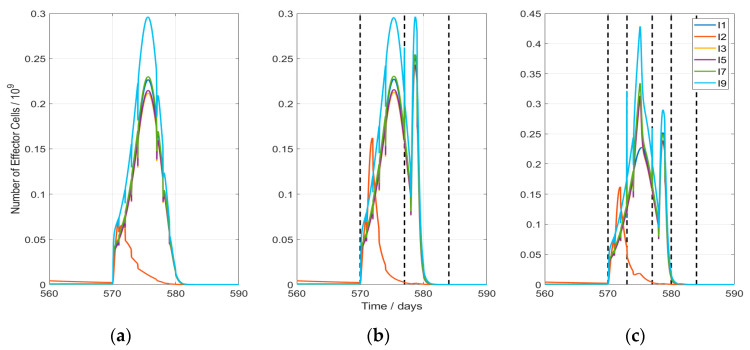
Development of effector immune cells during RT and HT-RT (in case of HT-induced modification of all parameters, according last column in Table 4). The impact of every RT fraction (5 fractions in the first week starting at day 570 and 4 of 5 fractions of the 2nd week starting at day 577) on the effector cells is visible as a spike-shaped drop of the cell number. The scenarios presented here correspond to (**a**) Case with no HT (RT1HT0); (**b**) Case RT1HT3; (**c**) Case RT1HT2. A dashed line is plotted each time a HT session is performed.

The immune response after RT is only produced during the first 10 days of treatment (Figure 5). This explains why the hyperthermia treatment HT2, which is the one with more hyperthermia sessions during those days, results in the highest TCP value. Additionally, spikes are visible at the position of each RT fraction because of the radiation-mediated effector cell elimination. On the other hand, rises in the effector cell production are visible after each hyperthermia session (Figure 5b,c): one just after HT produced by the perfusion like effects and another one 1–2 days after because of slow processes. In this figure, it is also observed that the anti-host immune response after RT is augmented by HT.

## 4. Discussion

As stated in the introduction, the results of this study cannot be applied directly to clinical treatments since they represent the behavior of an artificial system. Besides the uncertainty of many of the used parameters, a very simplistic description of the immune system is used. One of the main shortcomings is the lack of an immunological memory. In addition, only the local response in the tumor compartment is regarded. The anti-host immune reaction observed in our simulations may be interpreted as a local inflammatory process after radiation. In the case of additional compartments containing only non-irradiated neighboring host tissue, the training of the perceptron may result in different weights for host tissue and a subsequent modification of the anti-host response. Regarding this aspect, a multi-compartmental model would be closer to the real patient.

In addition, the inclusion of HT in the model follows simplistic concepts leading to the question of whether they are appropriate. In particular, thermo-tolerance is not considered. Therefore, the conclusions may not be appropriate for shorter intervals of HT sessions at higher temperatures (above 41–42 °C). Interestingly, the influence of variations in the HT sub-models does not lead to large differences in the outcome. This can be seen as an indication that—at least for larger time scales—the dynamic interplay between the adaptive immune system (perceptron training) and tumor-host ecosystem may be more important, independently of the details of the different sub-models.

The analysis of infiltrating immune cells in biopsy material can be compared to the time course of the effector cell populations in the model. The problems of comparing the model with such real-world data derived by biopsy material of cancer patients are manifold. The analysis of tumor samples by Holl et al. [57] revealed a percentage of overall lymphocytes of 2–39% of totally living cells. Not all of these cells can be considered as effector cells in the sense of our model. Therefore, it can be expected that the number of effector cells acting against the tumor should be clearly below (in the presented simulations, a percentage of 0.1–0.5% can be observed). Real world data give an indication for an upper limit (the simulation results are clearly below this limit) but also exhibit a large variation of patient—and tumor—specific responses.

Besides the percentage of effector cells in the peak of the immune response, a comparison of the production/invasion and elimination speed with real-world data would be interesting. According the work of Krosl et al. [58], the immigration (infiltration) of cells of the innate immune system seems to be very fast: neutrophils peak around 5 min and mast cells exhibit a pronounced rise during the first 25 min after Photodynamic Therapy (PDT) in CH3/HeN mice with implanted squamous cell carcinoma. No lymphocytes have been observed during the first 8 h after PDT. In our simulations, the effector cell number rise after the first RT fractions with a delay of 1 h at a high rate during 2–3 h followed by a slower increase over days. Regarding the point that these effector cells are part of the adaptive immune system, a slower process compared to the innate immune response can be expected, although the artificial immune system in our simulations was pre-exposed to the tumor antigens prior to the first RT fraction by necrotic tumor cells.

A stringent comparison with clinical trials is at the moment not possible and would require a sufficient number of patients in the different HT-RT treatment schemes (HTxRTy). A coarse indication may be obtained by a comparison with a clinical trial including patients with UICC stage I-IV anal cancer who received chemo-radiotherapy [55]: 50 out of the 112 patients received additional hyperthermia treatments. After 5 years follow-up, the overall response was significantly increased in the hyperthermia group (95.8 vs. 74.5%). The local recurrence-free after 5 years follow-up was 97.7% (HT) vs. 78.7% (no HT). These values are in agreement with the presented results. It is important to note here that only the case without HT (RT only) was adjusted to a TCP of 0.8. The fact that a comparable impact of HT, as observed by Ott et al. [55], was reached is based on the HT models used in the simulations.

During this study, a large number of simulations with varying conditions and parameter values have been executed (not shown). Over a large range of different parameter values, similar behavior of the system was observed. In this light, the semantic approach used for modelling in this study leads to the observation of some fundamental dynamic patterns which may allow general conclusions concerning the basic dynamics in such systems. However, the following conclusions are more or less restricted to the investigated scenarios and the proposed artificial immune–tumor ecosystem.

## 5. Conclusions

For the first time, a simulation for investigating the effect of a full HT-RT treatment on an artificial adaptive immune–tumor ecosystem is presented. In the investigated scenarios, RT leads to an anti-tumor as well as an anti-host response during RT. This effect is—especially for tumor cells—increased by the application of HT prior to selected RT fractions. The main effect of HT (Δ*TCP* = 0.13–0.18) is based on the adaption of the radio-sensitivity coefficients indicating a pivotal role of heat-induced, intra-cellular modifications. Perfusion or heat-based effects on the immune system seem to contribute less (Δ*TCP* = 0.003 − 0.019) in the investigated system. In addition, the influence onto the TCP between the two RT fractionation schemes is very small (Δ*TCP* = 0.001 − 0.011) and the RT2-fractionation turned out to be slightly less effective, in contrast to the findings by Scheidegger and Fellermann [59]. Even for the different HT protocols, the main rise of TCP is achieved by the early HT sessions. This is the reason why the HT2-protocol (as used for the HYCAN trial) exhibits a slightly better response. This is based on the fact that at the beginning of the therapy, more tumor cells are present and the effect of radiation-induced cell killing and immune activation is therefore stronger. As a possible consequence for clinical treatments, more HT sessions at the beginning of a HT-RT treatment seems to be favorable, as long as no thermo-tolerance will be induced.

During RT and HT-RT, a pronounced immune response contributes to tumor cell elimination by activation of the immune system via the perceptron response (rise of perceptron weights). As the tumor regrows after treatment, the secondary (late) immune response remains weak in all simulations and no radiation—or heat—induced anti-tumor vaccination effect was observed. The perceptron weights for host tissue evolve during the regrowth phase into negative values. This leads together with the decreased weights for the different tumor sub-clones to an immune-suppressive effect. This effect is based on the dynamic interplay between population (re-) growth and the perceptron training. If the immune system in patient would behave similar, this effect would be added to other effects based on the immune-suppressive strategies of tumor cells such as the release of immune-regulatory cytokines or changes in the microenvironment [60]. In general, the therapy outcome is strongly influenced by the combination of ecosystem dynamics and perceptron training. By implementing an immunological memory in the model, it would be interesting to search for scenarios where HT enhance or induce a memory-based anti-tumor response (HT-induced anti-tumor vaccination).

As a more general conclusion, a stringent and systematic comparison between the presented simulation and clinical trials requires trials with sufficient patients receiving treatments using similar fractionation schemes and with a careful documentation/reporting of achieved temperature courses during treatments (and time gaps between HT and RT).

## Figures and Tables

**Table 1:** Model parameters for ecosystem interactions: parameters used for the investigated scenario; parameters considered as susceptible for hyperthermia are indicated by an asterisk. Parameters are normalized to 10^9^ cells.

Parameter/Unit	Description	Default Value
$k_{T11}=k_{Tik}$/d^−1^	tumor growth rate constant	3 × 46 × 10^−2^
$k_{mut}$/d^−1^	mutation rate constant	10^−3^
$k_{eT}$/d^−1^	tumor cell elimination rate constant	4 × 10^−3^
$k_{TT}$/d^−1^	tumor cell growth inhibition	10^−4^
$k_{IT}$/d^−1^	immunogenic tumor cell elimination	1
$k_{HT}$/d^−1^	host-tumor cells interaction	10^−5^
$k_{TH}$/d^−1^	tumor-host cells interaction	2 × 2 × 10^−4^
$k_{aH}$/d^−1^	host cell growth	3 × 10^−2^
$k_{bH}$/d^−1^	host cell growth inhibition	1 × 2 × 10^−4^
$k_{eH}$/d^−1^	host cell elimination	10^−5^
$k_{pn}$/d^−1^	necrotic transformation rate constant	0 × 5
$k_{n}$/d^−1^	necrotic cell elimination	5
$k_{ap}$/d^−1^	apoptosis rate constant	2
$k_{IH}$/d^−1^	immunogenic host cell elimination	1
$k_{I}$/d^−1^	immune cell production and migration *	10
$k_{eI}$/d^−1^	immune cell elimination	1

**Table 2:** Model parameters for pattern-recognition: parameters used for the investigated scenario; parameters considered as susceptible for hyperthermia are indicated by an asterisk.

Parameter/Unit	Description	Default Value
$Y_{act}$	danger signal activation level	3
*ξ*	power of perceptron response function	9
$X_{act}$	pattern recognition level *	2
η	pattern presence weight for pre-necrotic cells	0.5
χ	pattern presence weight for necrotic cells	0.2
$L_{act}$	danger signal param. (Equation (7)) *	3
*a*/1 × d^−1^	perceptron learning rate	5

**Table 3:** Model parameters for radiobiological model: parameters used for the investigated scenario; parameters considered as susceptible for hyperthermia are indicated by an asterisk.

Parameter/Unit	Description	Default Value
$\alpha_{T}$/Gy^−1^	radiation sensitivity coefficient tumor cells *	0.28
$\beta_{T}$/Gy^−2^	radiation sensitivity coefficient tumor cells *	0.05
$\alpha_{H}$/Gy^−1^	radiation sensitivity coefficient host tissue	0.05
$\beta_{H}$/Gy^−2^	radiation sensitivity coefficient host tissue	0.01
$\alpha_{I}$/Gy^−1^	radiation sensitivity coefficient immune cells (effector cells)	0.1
$\beta_{I}$/Gy^−2^	radiation sensitivity coefficient immune cells (effector cells)	0.01
$\gamma_{T}$/d^−1^	radiobiol. repair constant for tumor cells	3
$\gamma_{H}$/d^−1^	radiobiol. repair constant for host tissue	10
$\gamma_{I}$/d^−1^	radiobiol. repair constant for immune cells	2
*R*/Gy/min	radiation dose rate	0.14

**Table 4:** TCP values after RT and HT-RT for the different combination of varying parameter values: $k_{I}$ is assumed to be perfusion-limited only (fast process only); the column “all parameters” shows the combined effect of all parameter values modified by HT. Protocols according to Figure 2.

Protocol	$\alpha_{T},\beta_{T}$	$\alpha_{T},\beta_{T},k_{I}$	$\alpha_{T},\beta_{T},X_{act}$	$\alpha_{T},\beta_{T},L_{act}$	All
RT1HT0	0.798 ^2^	no HT	no HT	no HT	no HT
RT1HT1	0.933	0.934	0.935 (0.933) ^1^	0.952 (0.935) ^1^	0.960
RT1HT2	0.979	0.980	0.980 (0.979) ^1^	0.988 (0.980) ^1^	0.990
RT1HT3	0.980	0.980	0.981 (0.981) ^1^	0.986 (0.980) ^1^	0.988
RT2HT0	0.801 ^2^	no HT	no HT	no HT	no HT
RT2HT1	0.931	0.932	0.931 (0.931) ^1^	0.951 (0.931) ^1^	0.951
RT2HT2	0.979	0.979	0.980 (0.979) ^1^	0.983 (0.979) ^1^	0.984
RT2HT3	0.979	0.979	0.980 (0.980) ^1^	0.981 (0.979) ^1^	0.982

^1^ Fast process (perfusion-limited modifications) for $X_{act}$ and $L_{act}$; ^2^ No HT applied.

## Data Availability

Program code is available on request to scst@zhaw.ch.
